# Assessment of the Influence of Protective Polymer Coating on Panda Fiber Performance Based on the Results of Multivariant Numerical Simulation

**DOI:** 10.3390/polym15234610

**Published:** 2023-12-03

**Authors:** Anna A. Kamenskikh, Lyaysan Sakhabutdinova, Yulija A. Strazhec, Anastasia P. Bogdanova

**Affiliations:** Department of Computational Mathematics, Mechanics and Biomechanics, Perm National Research Polytechnic University, 614990 Perm, Russia; lyaysans@list.ru (L.S.); strazhecjulia@gmail.com (Y.A.S.); anstasia_pankova@mail.ru (A.P.B.)

**Keywords:** polarization-maintaining fiber, Panda fiber, polymer, protective coating, geometric configuration, material behavior models, deformation behavior, birefringence, contact parameter, thermoviscoelasticity

## Abstract

This article considers the deformation behavior of Panda optical fiber using different models of material behavior for the tasks of predicting residual stresses after drawing when cooling from 2000 °C to room temperature (23 °C) and indenting the fiber into an aluminum half-space at different parameters. These studies were conducted for single- and double-layer protective coatings (PCs), at different values of external load and thickness of single-layer PC. This paper determined the fields of residual stresses in the fiber formed during the drawing process. They are taken into account in modeling the fiber performance in the further process of this research. This article investigated two variants of PC behavior. The influence of behavior models and the number of covering layers on the deformation of the “fiber-half-space” system was analyzed. This paper establishes qualitative and quantitative regularities of the influence of the external load magnitude and relaxation properties of PCs on the deformation and optical characteristics of Panda optical fiber.

## 1. Introduction

### 1.1. Research Objectives

The aim of this work was to investigate the deformation and contact parameters of Panda optical fiber based on the results of multivariate numerical simulation.

To achieve this goal, the following tasks were set:

1. Influence analysis of the effect of optical fiber material behavior models on technological residual stresses during workpiece cooling.
2. Influence analysis of the effect of quartz glass and material behavior models on deformation and contact parameters of Panda fiber during its indentation into aluminum half-space.
3. Influence analysis of the effect of load, in the presence of one layer of protective coating, on deformation, optical and contact parameters of the system “fiber-half-space” during indentation.

### 1.2. Problem Context

Optical fiber is a rather new material. However, nowadays it is difficult to imagine various types of infrastructure without its use. A wide variety of applications have been found for optical fiber for one human activity or another. In particular, it is a key element in modern telecommunication systems and is widely used to transmit data over long distances with high speed and minimal losses [1,2,3,4]. Optical fiber is often used in sensor manufacturing to measure strain [5], applied loads [6], stress [7], magnetic field and temperature [8], etc. [9,10,11,12]. It has made life easier as it allows rapid assessment of the durability not only of structures [11] but also of the human body [9].

Fiber optic cable consists of a core of silica glass, which itself is brittle, and various numbers of layers of protection. Polymer is a popular material for the fabrication of its protective coating. It can increase the strength of fiber optic cable, and provide thermal protection, corrosion protection, etc. It also has low weight and is easy to apply to the surface.

Various types of optical fibers exist today: bowtie, elliptical, pseudo-rectangle, elliptical core, panda [13,14] and others [15,16,17]. However, the process of developing improved types of designs does not stand still [18,19,20]. All types of optical fibers are multilayer structures. If the surfaces are not bonded, friction occurs between them, which also affects the durability of the fiber optic cable.

The study of tribological properties of materials has been and remains a relevant topic for scientists in various fields [21,22,23,24,25,26,27,28]. In order to reduce the level of friction force magnitude, various modifications are introduced in the material composition [24,25,26], lubricants are used [27], etc. [28]. Friction force and wear depend on surface roughness; thus, its processing has a significant impact on the deformation behavior of all surfaces in contact interaction. This also has an impact on the durability of the structure as a whole. The surface roughness of a material or structure depends on the type of its creation and processing [29,30].

A significant amount of research has been carried out on the operation of optical fiber, its manufacturing technology, etc. One important area of research is the detection of optical fiber faults in real time during operation [31], since fast response and accurate detection have good financial significance to support the design. However, a lack of design performance data has been reported by manufacturers [32].

The following parameters must be considered when investigating the performance of optical fiber: a large set of geometric characteristics, residual stresses accumulated from its manufacture (note residual stresses), surface roughness, tension force, surface interactions of the structure, etc. All these parameters affect the performance and durability of a structure to a greater or lesser extent. It is especially important to study them not as individual elements but the whole system together, taking into account their optical and deformation characteristics, material rheology, etc. [33,34]. Studies of the properties of optical fibers taking into account PC are also relevant [35,36]. This will help with obtaining a complete picture of understanding of the performance of optical fiber under different conditions of its operation. It will also allow more accurate predictions of its durability, light conductivity, longevity, etc. However, most of the studies are aimed at considering individual parts of the structure [4,16,37,38,39,40], and there are also studies in which only the light-conducting properties are determined [41].

### 1.3. Problem Description

Various residual stresses arise in the structural elements of specialized optical fibers during the manufacturing process. Initially, the residual stress field is formed during preform formation. This occurs during the manufacture of the light conductor and power rods. However, their level can be significantly reduced at the stages of jacketing (increasing the thickness) of the light conductor and etching off the outer layers of the power rods. The drawing process of the preform into fiber occurs after installing power rods into the workpiece. The residual stress field in Panda optical fiber is formed due to the incompatibility of temperature deformations when cooled from 2000 °C to room temperature during the drawing process. Residual stresses create conditions for maintaining the polarization of light in the light conductor due to the photoelastic effect. Obtaining data on the distribution of technological stresses is necessary not only for assessing the strength and optical characteristics of fibers but also to predict their behavior when used in various designs.

In this article, the basic problem is considered, but it helps to take into account certain features of the structure and to pass to the solution of a multi-parameter problem. Some of the parameters that affect the residual deformations, the degree of wear during operation, light conductivity, durability of the structure, etc., are the tension force of the optical fiber, the type of quartz glass surface treatment, the geometric characteristics of the cable, etc.

This work is aimed at the numerical modeling of Panda optical fiber. Also, different models of material behavior are implemented: thermoelastic and thermoviscous elastic. The research allows us to obtain quantitative and qualitative fundamental regularities, which in the future will form the basis for the production of real structures.

## 2. Materials and Methods

### 2.1. Optical Fiber Models for the Study of Stress–Strain State

**Figure 1:**
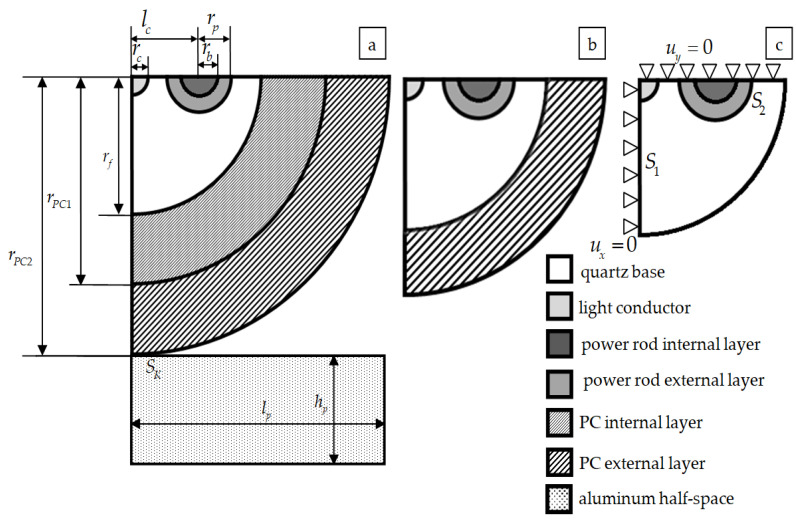
Design scheme of 1/4 cross-section a fiber: (**a**) with a two-layer protective coating and half-space; (**b**) with a single-layer protective coating; (**c**) without coating with boundary conditions.

This paper deals with two problems of deformation behavior of optical anisotropic Panda fiber. The first problem is the study of optical fiber with regard to single- and double-layer coatings during indentation into an aluminum half-space (Figure 1a). The second task is determining the residual stress fields occurring in the Panda optical fiber blank after drawing when cooling from 2000 °C to room temperature (23 °C). Figure 1c shows the considered calculation scheme and the boundary conditions used in modeling the cooling of the optical fiber blank.

Panda type optical fiber model: quartz base of SiO_2_ (mat. 1); light conductor (mat. 2) is quartz-doped GeO_2_; power rod internal layer (mat. 3) is quartz-doped B_2_O_3_; power rod external layer (mat. 4) is quartz-doped B_2_O_3_ and P_2_O_5_; PC internal layer made of polymer material (PC1) (mat. 5); PC external layer made of polymer material (PC2) (mat. 6). Polymer materials of PC internal and external layer differ in properties. The fiber is mated to the polished aluminum surface of the half-space.

The influence problem of fiber interface with metallic and non-metallic contact surfaces is relevant from the point of view of deformation characteristics analysis of the study object. The ideal contact is realized by means of creating a node-to-node mesh at the interface between the fiber and the protective multilayer coating and between the layers of the protective coatings. The contact is realized by means of a special contact pair of elements at the contact boundary $S_{K}$ between the fiber in the PC and the aluminum half-space.

The construction of the parameterized optical fiber model is based on the calculation scheme (Figure 1). Table 1 presents the values of the parameters that were considered in this paper.

Within this study, the total coating thickness $h_{PC}$ varies from 10 to 50 μm. The range includes the total thickness of the two-layer PC shown in Figure 1b and Table 1. The polymer-covered fiber is indented into the aluminum half-space with a constant force of 0.05 mN. The material behavior of the PC is considered in two statements: within the framework of elasticity theory; taking into account the viscosity of the polymer (thermoviscous elasticity).

### 2.2. Materials

**Figure 2:**
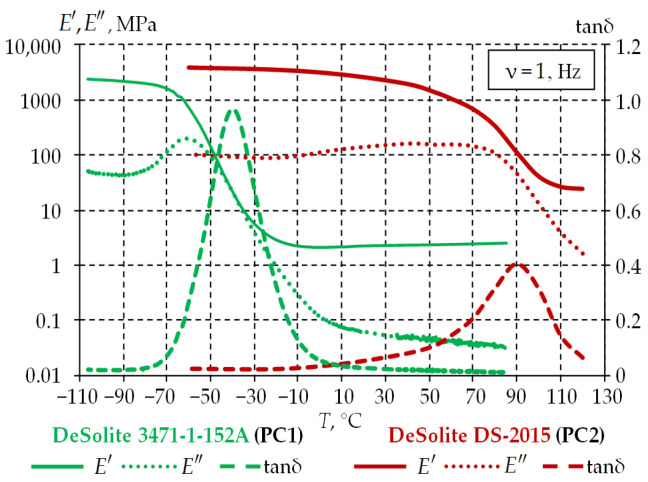
Dependences of PC polymer parameters *E′*, *E″* and tanδ on temperature.

The data obtained by the authors [42] during the study of UV polymers from DSM Desotech, produced under the DeSolite trademark, were used in this article. The first layer, PC1, is made of DeSolite 3471-1-152A, and the second layer PC2 is made of DeSolite DS-2015. The experimental study was carried out on film samples on a TA Instruments DMA Q800 dynamic mechanical analyzer in the temperature range [−110; +130] °C at a harmonic disturbance frequency of 1 Hz and a heating rate of 2 °C/min. The temperature dependences for the elastic modulus *E′*, the loss modulus *E″* and the mechanical loss tangent tanδ were obtained as a result of the study. They are presented in Figure 2.

The generalized Maxwell model was chosen to describe the thermomechanical behavior of PC. It is implemented in the ANSYS Mechanical APDL package using the approximation of relaxation functions by exponential Prony series. The mathematical formulation and the obtained material constants are given in the articles [43,44].

Optical fiber consists of quartz glasses, which differ by the presence or absence of alloying additives. Glass is subject to relaxation transitions, like polymers, when heated or cooled. A material changes state from solid to viscous or from viscous to solid within a certain temperature range. But such a transition associated with a change in rigidity is not phase, because quartz glass is an amorphous material [45]. A mathematical model based on the Anand model was previously developed to describe the behavior of structures under such conditions [46]. The model allows one to take into account the entire history of temperature changes, the cooling rate, the glass transition and relaxation processes characteristic of structures made of vitrified materials.

The behavior of doped silica glass was described using a Maxwell-type linear viscoelasticity model with viscosity. The approach was implemented in the ANSYS Mechanical APDL application software package using the Anand model [47]. Dependences of viscosity, thermal expansion coefficient, Poisson’s ratio and elastic modulus on the percentage of alloying additives B_2_O_3_, P_2_O_5_ and GeO_2_, obtained by approximating experimental data from literature sources, are used to describe the material behavior of structural elements (light conductor, power rods) [48,49].

Thermoelastic models of the structural material behavior are considered to conduct a comprehensive comparative analysis. The parameter values correspond to the instantaneous characteristics of materials for automating computational procedures. Table 2 presents the elastic properties of the materials. Materials 1–4 are quartz glass and are characterized by the presence and content of alloying additives, which have a significant influence on physical–mechanical and thermomechanical properties of the materials. Materials 5–6 are polymers and are used as soft buffer intermediate or primary protective coating (internal PC is DeSolite 3471-1-152A) and external hard protective coating (external PC is DeSolite DS-2015). The mating surface is an aluminum half-space (mat. 7), which allows one to simulate the mating with an aluminum coil in the primary test of the final product (Panda fiber). Material 6 (DeSolite DS-2015) was selected as the PC material for the model with one layer of PC.

The viscoelastic behavior of PC materials is modeled using a Prony series describing the Maxwell model [43]. The temperature dependence of material properties is described using the Williams–Landel–Ferry (WLF) temperature–time analogy [50]. The main parameters of the viscoelastic PC model are given in [44].

Cooling of the fiber during drawing begins at a temperature of 2000 °C [51]. The use of fictitious temperatures to describe the initial cooling point of the Panda glass preform is necessary if we are to describe its solid behavior [52]. These fictitious temperatures are 1450–1600 °C. Taking into account the fictitious temperatures of the initial cooling point of the workpiece is necessary in the elastic problem formulation. Initially, the Anand model was intended to describe metal melts and their transition from liquid to solid and vice versa [53,54]. Using the Anand model adapted for glass allows one to simulate residual stresses from the actual cooling temperature of the workpiece.

### 2.3. Fiber Optical Parameters

Material and mode birefringence are the main optical characteristics of fibers. There are a number of works that propose an analytical evaluation of birefringence for Panda fiber [55,56,57]. The transition formulas from stress–strain parameters to optics described in the open literature were used to estimate the optical characteristics of the system.

The mode birefringence according to [55] can be calculated analytically, through the geometrical parameters of the optical fiber and material constants by Formula (1):

(1) $$B=\left(-4\cdot C\left(\lambda \right)\cdot E\cdot \Delta \alpha_{s}\cdot \Delta T_{s}\cdot r_{c}^{2}\right)/\left(l_{c}^{2}\left(1-\nu \right)\right)$$

where $C\left(\lambda \right)=3.36\times 10^{-12}$ Pa^−1^ is the photoelastic constant for wavelength λ=1.55 (it is calculated from the photoelastic coefficient $C_{1}=-6.5\times 10^{-13}$ Pa^−1^, $C_{2}=-4.22\times 10^{-12}$ Pa^−1^), E is the Young’s modulus of material 4, v is the Poisson’s ratio of material 4, $r_{c}$ is the radius of the power rod, $l_{c}$ is the distance between the centers of the power rod and the fiber, $\Delta T_{s}=-700$ K is the difference between the softening temperature of the power rod material and the quartz shell, $\Delta \alpha_{s}=\alpha_{SAP}-\alpha_{mat4}$ is the difference between the thermal expansion coefficients of the materials of the power rod and the quartz shell, $\alpha_{SAP}$ is the average coefficient of thermal expansion of the power rod material calculated according to the volume fraction of materials 2 and 3, and $\alpha_{mat4}$ is the coefficient of thermal expansion of material 4.

The material birefringence of numerical calculations is determined by the formula:

(2) $$B_{m}=\left(C_{1}-C_{2}\right)\left(\sigma_{x}-\sigma_{y}\right)$$

The mode birefringence of numerical calculations is determined by the formula:

(3) $$B=\left(C_{1}-C_{2}\right)\int_{S_{C}}\left(\sigma_{x}-\sigma_{y}\right){\left|E^{*}\right|}^{2}dS_{C}/\int_{S_{C}}{\left|E^{*}\right|}^{2}dS_{C}$$

where $E^{*}$ is the intensity distribution of the main mode over the cross-section of the fiber, $S_{C}$ is the cross-sectional area of the core, and $\sigma_{x}$, $\sigma_{y}$ are the components of the stress tensor.

### 2.4. Numerical Finite Element and Methods

Numerical modeling is implemented in the finite element method package ANSYS Mechanical APDL 2021R2 (Livermore, CA, USA). The finite element mesh, which was created using iso-parametric planar three-node elements of the 1st order of approximation with two unknowns in each node, is Plane182. Special elements Conta171 and Targe169 are used to realize surface-to-surface coupling in the PC–half-space contact zone. The problem was considered without taking into account friction at the fiber and half-space interface.

This paper considers 7 variants of finite element mesh (FEM) to evaluate the influence of the degree of discretization of the computational domain on the numerical solution of the workpiece cooling problem. The overall size of the finite element in material volumes 1–4 is parameterized by the minimum overall size of the model—the radius of the light-conducting core $l_{e}=r_{c}/k$, where $l_{e}$ is the linear size of the finite element, and k is the number of partitions in the range from 2 to 15.

In the second stage, this paper investigated the effect of the FEM of the contact area on the contact pressure under constant indentation force and under elastic behavior of all materials of the contact assembly without taking into account the friction on the PC–half-space interface surface. In the elastic formulation, the size of the area on which the contact elements are applied is $4\cdot r_{c}=12$ μm. The size of the elements in the contact region is also tied to the minimum overall size of the structure $l_{S_{K}}^{e}=r_{c}/k$, where $l_{S_{K}}^{e}$ is the linear size of the terminated element, and k j is the number of partitions in the range of 5 to 50. For the viscoelastic model, the size of the area where the contact elements are mapped was increased $15\cdot r_{c}=45$ µm. In the contact region, the finite element size is reduced, and the FE size increases gradiently moves farther away from the contact region.

Based on the obtained data on the distribution of stresses in the workpiece, this paper found that the minimum difference in the results of the numerical solution of the problem with a significant increase in the counting time is achieved at the element size $l_{e}=r_{c}/12$. The introduction of nonlinear models of material behavior into the computational scheme leads to an increase in computational time, including in the simulation of the iterative procedure for finding the contact surface in the fiber–half-space interface zone, and then FEM with 33.05 thousand node unknowns for discretization of quartz glass material volumes was chosen as a rational one, which corresponds to the finite element size $l_{e}=r_{c}/10$.

In the fiber–half-space interface zone, a finite element mesh with a maximum number of 181 contact elements was selected with an initial contact element area of 12 µm, which corresponds to the contact finite element size $l_{S_{K}}^{e}=r_{c}/30$.

When the thermoviscous elastic behavior of polymers is taken into account, the radius of the contact element area is increased to 45 μm, with the size of the contact elements equal to $l_{S_{K}}^{e}=r_{c}/30$, as in the elastic behavior of PC materials.

The contact pressure is less sensitive to the degree of discretization of the system in the model, taking into account the viscosity of the materials of the gears. It is noted that the distribution is not canonical, and the maximum is shifted relative to the point of initial contact. The study of the influence of finite element partitioning taking into account the viscosity of polymer PC showed that the solution with the selected finite element size (450 finite elements at 45 μm) allows one to achieve convergence of the numerical solution of the problem. A further increase in the degree of discretization of the system does not significantly change the nature of the distribution and the level of contact pressure with a significant increase in the time of calculations.

Thus, the rational size of the finite element in the PC–half-space contact area for all variants of the problem formulation is equal to $l_{S_{K}}^{e}=r_{c}/30$.

## 3. Results

### 3.1. The Influence Investigation of the Effect of the Glass Material Behavior Model on the Residual Stresses during Cooling of the Workpiece

This paper considered two glass behavior models for predicting residual stress fields in a Panda optical fiber blank. Modeling of glasses as an elastic body is considered in comparison with the Ananad model, which takes into account the dependence of viscosity on temperature.

**Figure 3:**
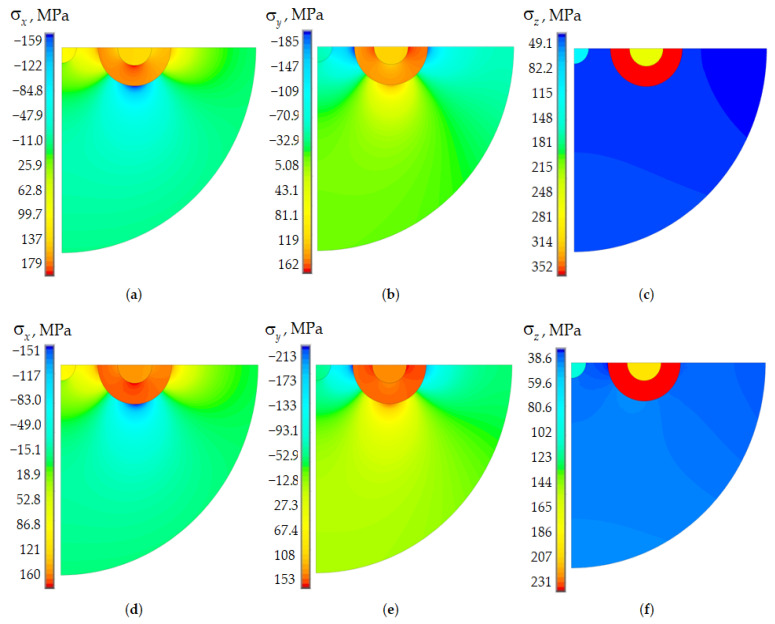
Residual stresses of Panda optical fiber (fictitious workpiece cooling start temperature of 1600 °C): (**a**–**c**) elastic body; (**d**–**f**) viscoelastic body (Anand model); (**a**,**d**) $\sigma_{x}$; (**b**,**e**) $\sigma_{y}$; (**c**,**f**) $\sigma_{z}$.

Figure 3c presents the residual stress intensity fields in the fiber cross-section to evaluate the influence of doped glass behavior models on the deformation parameters of the structural elements during cooling of the workpiece. The results are presented for a fictitious temperature of the cooling start at 1600 °C for the workpiece. PC volumes are “killed” when simulating the workpiece cooling. They are “revitalized” only at the end of the technological cooling stage and are taken into account only in the indentation problem.

The distribution pattern of residual stress components has slight differences in the elastic and viscoelastic formulation. The levels of $\sigma_{x}$ and $\sigma_{y}$ differ little under different models for describing the behavior of glasses. The $\sigma_{z}$ component’s maximum level is more than 34% higher in the elastic setting. This fact can have a significant impact on optical characteristics. The residual stresses level is consistent with the data obtained in [58].

The mode birefringence found analytically for this fiber is $4.83\cdot 10^{-4}$ according to [44]. The mode birefringence obtained at a fictitious temperature at the beginning of workpiece cooling: $B=4.89\cdot 10^{-4}$ is elastic body; $B=4.69\cdot 10^{-4}$ is viscoelastic body. The fictitious temperature allows one to obtain the fiber birefringence with an error of 1.3 and 2.87% for the elastic and viscoelastic description of the materials, respectively, but this is at a temperature of 1600 °C.

**Figure 4:**
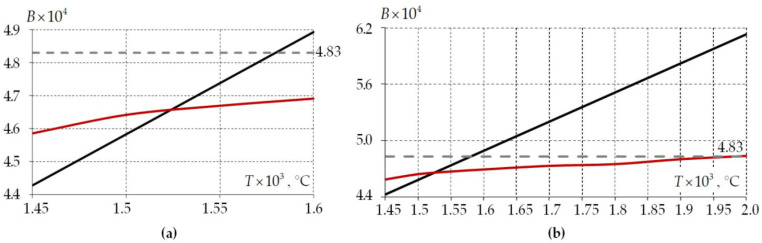
The mode birefringence’s dependence of the fiber on the cooling start temperature: (**a**) the fictitious temperatures region; (**b**) the region including the actual temperature of the cooling start; black line is elastic body; red line is viscoelastic body; the gray dashed line is an analytical solution.

The effect of fictitious temperature level on mode birefringence was investigated (Figure 4a). The capabilities of the Anand model for simulating glass cooling at process temperatures were also examined (Figure 4b). Modeling of glasses as an elastic body was performed in the region above 1600 °C to analyze the error of the material behavior elastic model.

Mode birefringence in the elastic formulation has a strong dependence on the temperature at which cooling begins. The dependence is linear. The numerically obtained value of B exceeds the analytical solution at temperatures above 1575 °C. The deviation of the residual stress fields has a significant effect on B when the cooling start temperature is below 1550 °C. For example, the mode birefringence deviation reaches 8.3% at a fictitious cooling start temperature of 1450 °C. The model is not applicable in temperature zones above 1600 °C. An elastic model of glass behavior is presented in Figure 4b to assess its use on optic effects at temperatures above 1600 °C. The optical characteristics deviation of the fiber from the analytics reaches more than 26% at the actual cooling temperature of 2000 °C. The elastic model of glass behavior can only be used at fictitious temperatures.

The Anand model allows one to take into account relaxation transitions in glass during cooling. This makes it possible to simulate the technological process at cooling temperatures above 1600 °C. Mode birefringence is lower than the analytical solution in the region of fictitious cooling start temperatures by 3–5%. It increases nonlinearly with increasing cooling start temperatures. The value of B strives to be analytical when tending to the actual cooling start temperatures when describing glass as a viscoelastic body.

**Figure 5:**
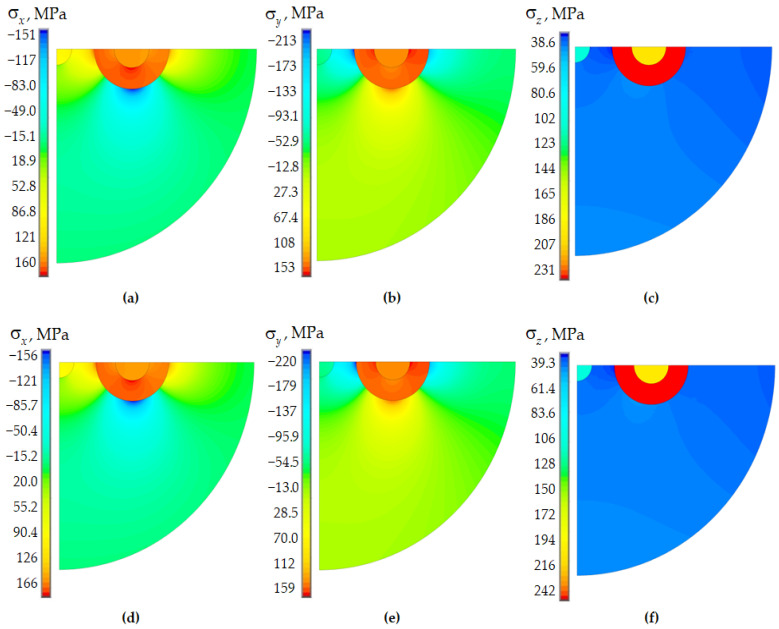
Residual stresses of Panda optical fiber (viscoelastic body, Anand model): (**a**–**c**) fictitious workpiece cooling start temperature 1600 °C; (**d**–**f**) actual cooling start temperature 2000 °C; (**a**,**d**) $\sigma_{x}$; (**b**,**e**) $\sigma_{y}$; (**c**,**f**) $\sigma_{z}$.

A distribution fields comparison of residual stresses is shown in Figure 5 with a viscoelastic model of glass behavior for fictitious and actual cooling start temperatures.

A significant increase in the cooling start temperature of the workpiece leads to a slight increase in the residual stresses level. This fact is associated with stress relaxation during the cooling process thanks to the Anand model. The residual stresses level largely depends on the starting temperature and the cooling rate. This model feature can lead to a significant deviation in the deformation and optical characteristics from the parameters of the real object under certain process conditions. Complicating the behavior model of glasses is one of the options for developing research.

**Figure 6:**
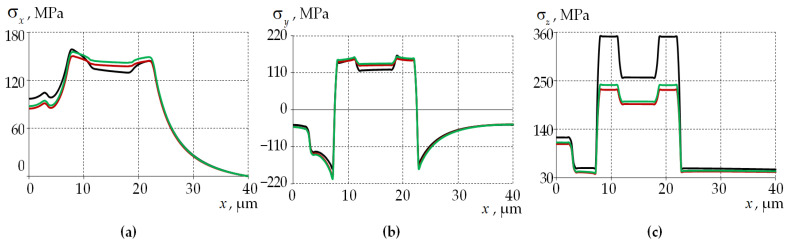
Diagrams of residual stresses along the central section of Panda fiber: (**a**) $\sigma_{x}$; (**b**) $\sigma_{y}$; (**c**) $\sigma_{z}$; the black line is elastic body at a fictitious cooling start temperature of 1600 °C; the red line is viscoelastic body at a fictitious cooling start temperature of 1600 °C; and the green line is viscoelastic body at the actual cooling start temperature of 2000 °C.

For a more detailed analysis, the distributions of the residual stress tensor components along the central cross-section of the optical fiber are shown in Figure 6. The results are presented for elastic and viscoelastic models of material behavior at a fictitious cooling start temperature of 1600 °C. The residual stresses distribution during the viscoelastic behavior of glasses with a cooling start temperature of 2000 °C is also shown.

The use of the thermoviscous elastic behavior model for glass materials with different doping has a significant effect on the values of the stress tensor components along the central section of the optical fiber. The use of this model allows one to reduce the stress drop near the interface zones of the materials. Modeling glass as an elastic body gives insignificant deviations in the $\sigma_{x}$ and $\sigma_{y}$ components at a fictitious cooling start temperature of 1600 °C. The main influence of the thermoelastic material model is observed in the $\sigma_{z}$ component in the power rods region.

Based on the results obtained, it is concluded that the consideration of the thermoviscous elastic behavior of glasses during cooling of the optical fiber blank significantly affects the distribution of deformation parameters. To obtain more accurate quantitative results of predicting residual stresses in the Panda optical fiber blank during cooling from 2000 °C to room temperature (23 °C), it is necessary to take into account the thermoviscous elastic behavior of the blank materials.

### 3.2. Comparative Analysis of Deformation Parameters of the Solution of the Fiber Indentation Problem with Single- and Two-Layer Polymer Coating under Different Models of Material Behavior

The problem of finding the stress–strain state of optical fibers in single- and two-layer polymer protective coatings (PCs) during indention in an aluminum half-space was solved for investigating the influence of behavior models on structural materials. The calculation scheme is presented in Figure 1a. The indentation force is constant at 0.05 mN. The problem was considered without taking into account friction at the fiber and half-space interface.

Four options for combining behavior models of glass and polymer PCs were considered: dc. 1 is the elastic behavior of glasses and polymers; dc. 2 is the viscous behavior of glasses and the elastic behavior of polymers; dc. 3 is the elastic behavior of glasses and the viscoelastic behavior of polymers; dc. 4 is the viscoelastic behavior of glasses and polymers.

A fictitious temperature of 1600 °C was chosen when simulating the cooling start of a glass workpiece in an elastic model of glass behavior. The actual temperature of 2000 °C was chosen when simulating the cooling start of a glass workpiece in a viscoelastic model of glass behavior.

The temperature for applying the PC is not disclosed as part of the technological process. The PC volumes of materials are modeled as “revitalization” of the finite elements at room temperature within the framework of the first approximation.

**Figure 7:**
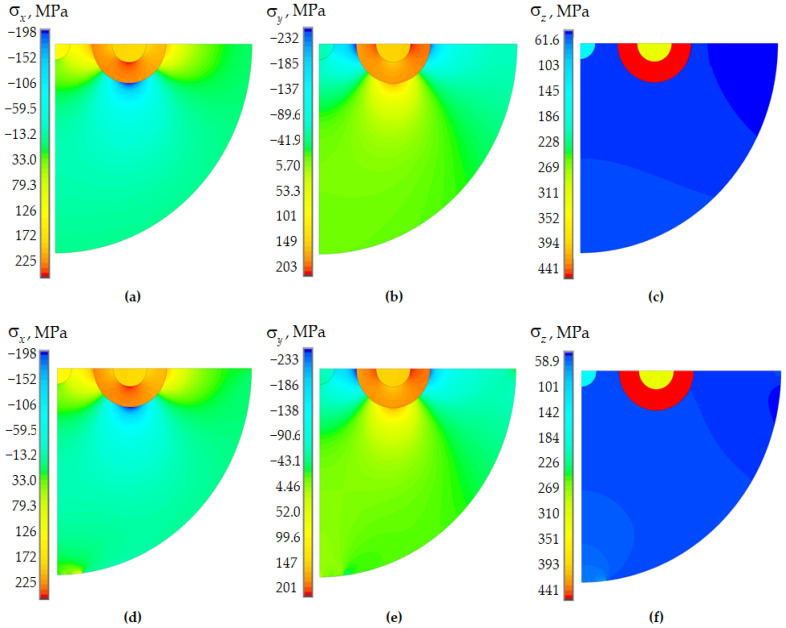
Components of the stress tensor during fiber indentation into an aluminum half-space and viscoelastic model of glass behavior: (**a**–**c**) elastic behavior of the polymers; (**d**–**f**) viscoelastic behavior of polymers.

The distribution of stress tensor component fields is shown using as, an example, dc. 2 and 4, with a two-layer PC in Figure 7. Data are shown after fiber indentation into the half-space.

The distribution fields of the stress tensor components are generally correlated with the residual stresses after cooling of the workpiece. The maximum levels of the stress tensor components are observed in the power bars. The increase in the level of the considered parameters is observed in the viscoelastic behavior of the polymer coating in the interface zone of the glass elements with the inner PC volume. This effect is connected with the following factors: the pressure of the inner layer of the protective coating on the quartz base; the absence of a contact interface between the inner PC and the quartz base, as well as between the PC layers; and the tendency of a part of the quartz glass volume to stretch along the *x*-direction due to polymer deformation.

The maximum stress level in the interface zone of the quartz base and inner PC under viscoelastic behavior of polymers was: $\sigma_{x}=129$ MPa, $\sigma_{y}=62.8$ MPa and $\sigma_{z}=91.1$ MPa. Absolute values of the stress tensor components reach: $\sigma_{x}=7.8$ MPa, $\sigma_{y}=0.73$ MPa and $\sigma_{z}=70$ MPa at the elastic behavior of polymeric materials in the quartz–PC interface zone. The stresses along the x and y coordinates are orders of magnitude lower for the elastic behavior of PC materials than for viscoelastic behavior.

The maximum level of stress components obtained in the process of fiber indentation into the half-space under elastic behavior of glasses is about 25% lower than under viscoelastic behavior. This fact can be directly related to the fact that the fictitious temperature at the beginning of cooling the workpiece in the elastic formulation of the problem is lower by 25% than the real one. The polymer material behavior model has a negligible influence on the maximum stress level in the glass elements.

The maximum stress and strain intensity levels in the PC material volumes have minimal dependence on the behavior models (Table 3).

This paper observes an insignificant influence of the glass behavior model on the stress and strain intensity of the polymer coatings (less than 1%). As expected, the maximum level of stress intensity is observed in the volume of the outer PC. $\max\sigma_{\mathrm{int}}$ is observed near the half-space contact zone in PC2 with the elastic behavior of the polymers. However, with the viscoelastic behavior of the polymers, $\max\sigma_{\mathrm{int}}$ is observed in the interface zone of the inner and outer protective layers in PC2. The maximum strain level is observed in the inner PC. In the viscoelastic behavior of polymers in the volume of PC2 material, it reaches values about ~36%, which has a significant effect on the stress–strain state of the silica fiber base and the outer PC.

$\max\sigma_{\mathrm{int}}$ are larger by 70% and smaller by 84% in the volumes of PC1 and PC2 materials, respectively, for the viscoelastic behavior of PC materials. $\max\varepsilon_{\mathrm{int}}$ are larger by more than 2 and 16 times for the viscoelastic behavior of PC1 and PC2, respectively. Consideration of the viscosity of the coating polymers has a significant effect on the deformation behavior of the fiber system in PCs when it is coupled with the aluminum half-space.

The next step of the research was to compare the deformation behavior of optical glass in single-layer and two-layer PCs when indented with constant force (Table 4). The thickness of the single-layer PC is equal to the total thickness of the two-layer PC $h=h_{PC1}+h_{PC2}=43.5$ μm.

For the elastic behavior of the glass elements, the maximum level of the stress tensor components is lower by more than 25% for single- and double-layer PCs.

It is also worth noting that this level is higher by no more than 1.2% in the model with single-layer PCs. The maximum differences are observed in the elastic behavior of the polymers in the $\sigma_{x}$ component of the stress tensor. This is due to the appearance of tensile stresses in the interface zone with the inner PC. This effect can be eliminated by introducing a contact pair of elements into the model with full adhesion conditions at the fiber–coating interface and between the coatings. The PC geometry has a minimal effect on the components of the $\sigma_{z}$ stress tensor in all combinations of material behavior models (no more than 0.15% and 0.03% for elastic and viscoelastic polymer behavior, respectively).

The single-layer polymer coating is modeled using PC2 material. Table 5 shows the level of influence of PC geometry on the following parameters: coating deformation; optical performance—from PC2 material at different combinations of material behavior models; at different model parameters; at single- and double-layer PCs.

The maximum intensity of stresses and deformations of the outer PC is lower in the single-layer geometry. This is due to the absence of the negative influence of the inner PC in the constrained state. In further studies, it is necessary to analyze the influence of the material of the inner PC on the behavior of the system in multilayer coating geometry.

Comparing the parameters obtained for single-layer and two-layer PCs, the following dependencies are observed: the maximum level of stress and strain intensity is on average 36% lower; the mode birefringence does not exceed 1%—in the case of a single-layer PC.

**Figure 8:**
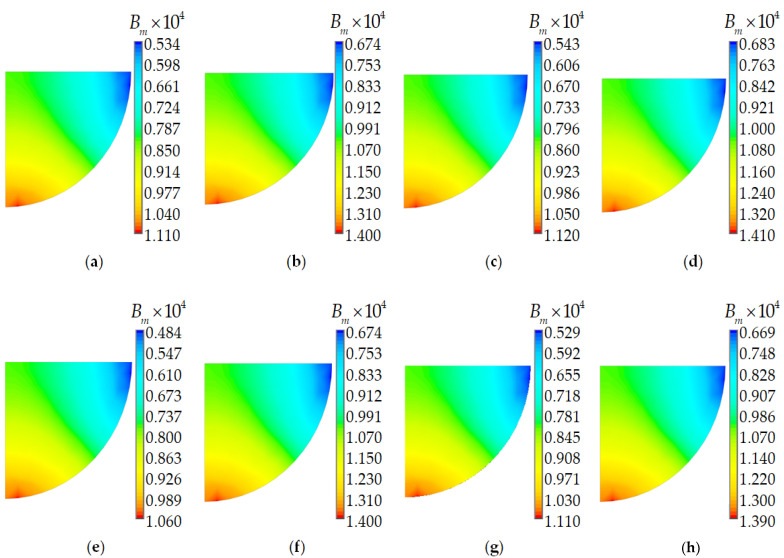
Material birefringence: (**a**–**d**) two-layer PCs; (**e**–**h**) single-layer PCs; (**a**,**e**) dc. 1; (**b**,**f**) dc. 2; (**c**,**g**) dc. 3; (**d**,**h**) dc. 4.

Figure 8 shows the character of the material birefringence distribution.

The distribution of material birefringence does not depend on the material behavior models. Its maximum value is observed in the lower part of the light conducting core. The level of material birefringence depends strongly on the glass behavior model and only slightly on the polymer behavior model. In general, the material birefringence is 32 and 25% lower for the elastic glass behavior combined with the elastic and viscoelastic polymer behavior models, respectively.

**Figure 9:**
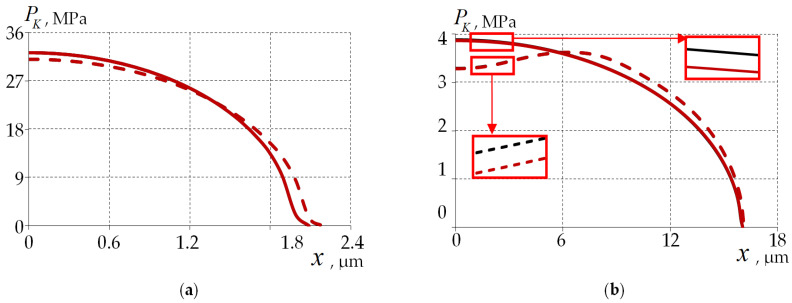
Distribution of contact pressure on the mating surfaces for the elastic (**a**) and the viscoelastic behavior PCs (**b**): the dashed line is two-layer PC; the solid line is single-layer PC; the black line is elastic behavior of glass; and the red line is viscoelastic behavior of glass.

Figure 9 evaluates the effect of the PC geometry and the combination of material behavior models on the contact pressure. The contact is modeled without considering the friction at the fiber–half-space interface in a first approximation. The main characteristics of the contact zone are the nature of the distributions and the level of contact pressure as well as the contact area.

The glass behavior model does not have a strong influence on the contact parameters: not more than 3% and 8% for the elastic and viscoelastic behaviors of polymers, respectively.

The geometry of the coating has an influence on the parameters of the contact area: the type and the maximum value of the contact pressure. For a single-layer PC, the following is observed: an increase in $\max P_{K}$ of 4% and 7% and a decrease in contact area of 4.5% and 1.2% for elastic and viscoelastic polymer behavior, respectively.

This paper observes a shift in the maximum relative to the center of the model in a multilayer coating due to deformation of the inner PC under the viscoelastic behavior of the materials. This effect is not observed for a single-layer PC. The character of the contact pressure distribution is close to the canonical form of the Hertz problem. Its nonlinear character influences the stress–strain state of the quartz substrate.

In further studies, it is necessary to take into account the contact in special elements located in the interface areas of the quartz base and the inner PC, as also between the layers of the PC. In addition, it is necessary to analyze the settings of the contact zone: full adhesion and frictional contact. The influence of the friction coefficient in all interface areas on the system performance is also analyzed.

### 3.3. Assessment of the Load Level Effect on the Behavior of Panda-Type Optical Fibers in Single- and Double-Layer Protective Coatings

Subsequently, effects of load level on fiber–half-space behavior were investigated for different PC geometries and material behavior combinations. All variants of the material behavior combinations (dc. 1–4) were investigated. However, the results are shown only for the glass viscoelastic behavior under elastic and viscoelastic behaviors of polymers. The load was varied from 0.01 to 0.1 mN.

**Figure 10:**
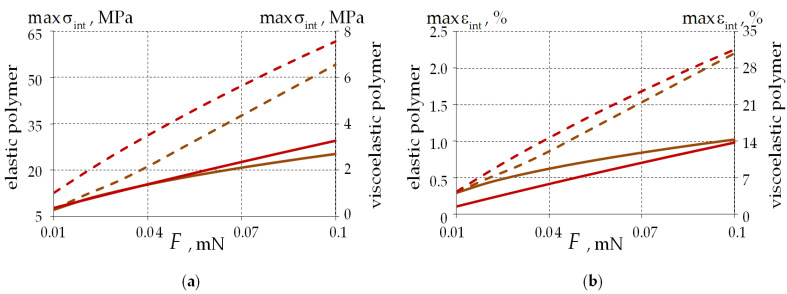
Dependence of the stress–strain state parameters in the PC2 material’s volume on the load: (**a**) the stress intensity; (**b**) the strain intensity; the dotted line is two-layer PC; the solid line is single-layer PC; the brown line is an elastic polymer; and the red line is a viscoelastic polymer.

Figure 10 shows the intensity of stresses and deformations in the external PC dependences on the acting load magnitude.

A nonlinear dependence of the stress–strain state parameters on load is observed. When the load is increased by a factor of 10, it is established that:

- Single-layer PC—increase in $\max\sigma_{\mathrm{int}}$ by 3.55 and 9.19 times, $\max\varepsilon_{\mathrm{int}}$ by 3.55 and 9.19 times with elastic and viscoelastic behaviors of PC2 material, respectively.
- Two-layer PC—increase in $\max\sigma_{\mathrm{int}}$ by 7.33 and 7.37 times, $\max\varepsilon_{\mathrm{int}}$ by 7.29 and 7.37 times for elastic and viscoelastic behaviors of PC2 material, respectively.

The viscoelastic behavior of the polymer has a significant effect on the system performance:

- $\max\sigma_{\mathrm{int}}$ is on average 90.17% and 84.82% smaller for single-layer and two-layer PCs, respectively;
- $\max\varepsilon_{\mathrm{int}}$ is on average 10.14 and 15.64 times larger for single-layer and two-layer PCs, respectively.

Under the elastic behavior of glass materials, the difference in the stress–strain state parameters of the system does not exceed 2% and 1% for single-layer and two-layer PCs, respectively.

The maximum level of stress and strain intensity is higher for two-layer PCs.

**Figure 11:**
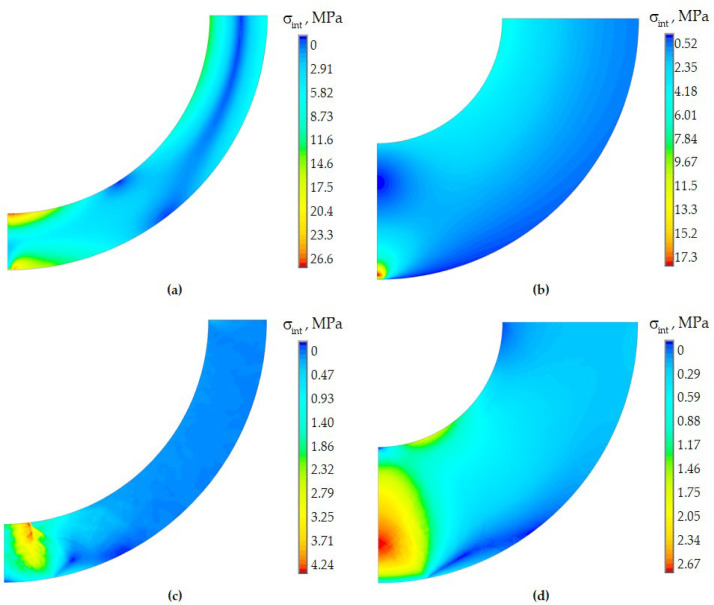
Stress intensity in polymer PC2: (**a**,**c**) two-layer PCs; (**b**,**d**) single-layer PCs; (**a**,**b**) elastic behavior; (**c**,**d**) viscoelastic behavior.

The internal PC affects the stress–strain state of the system and the contact zone in the case of multilayer pavement geometry. This geometry results in a zone of maximum stress near the interface of the PC layers. Figure 11 shows the stress intensity distribution fields in the volume of the PC2 material in contact with the aluminum half-space.

For single-layer protective coatings, the maximum stress intensity is observed near the contact zone. The area of maximum stress occupies a larger volume of the coating due to the viscoelastic behavior of the materials. However, the level of maximum stress intensity is orders of magnitude lower.

In the case of two-layer PCs, the area of maximum stress intensity is observed in the interface zone of the coating layers. In the elastic formulation, it is more uniform and does not have a strong influence on the deformation behavior of the quartz glass and the contact zone. In the viscoelastic setting, negative effects are observed due to the deformation behavior of the inner PC layer and its influence on the quartz base and the outer coating.

**Figure 12:**
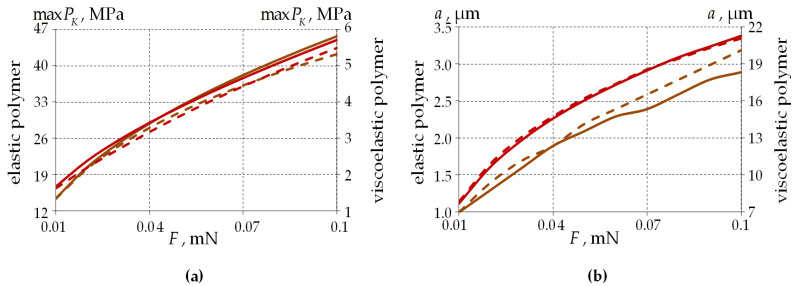
Contact parameters’ dependence on the load: (**a**) contact pressure; (**b**) the radius of the contact area; the dotted line is two-layer PC; the solid line is single-layer PC; brown line is an elastic polymer; and red line is a viscoelastic polymer.

Figure 12 examines the load effects on the contact parameters for single-layer and two-layer PCs.

When the load is increased by a factor of 10, the following is observed:

- Single-layer PC—increase in $\max P_{K}$ by 3.18 and 3.41 times, radius of contact by 2.89 and 2.77 times for elastic and viscoelastic behaviors of PC2 material, respectively.
- Two-layer PC—increase in $\max P_{K}$ by 2.95 and 3.38 times, radius of contact by 3.19 and 2.68 times for elastic and viscoelastic behaviors of PC2 material, respectively.

The viscoelastic behavior of the polymer has a significant effect on system performance:

- $\max P_{K}$ is on average 88% larger regardless of the PC geometry.
- The contact radius is on average 7.34 and 7.68 times larger for single-layer and two-layer PCs, respectively.

For the elastic behavior of the glass materials, the differences in contact parameters from the viscoelastic model are less than 1% for all PC geometries considered.

The maximum contact pressure level is higher for two-layer PCs. The contact area radius for the viscoelastic behavior of PCs has a small dependence on the coating geometry. The differences in the contact area radius for the elastic behavior of PCs are up to 9.5% for single-layer and two-layer PC geometries.

**Figure 13:**
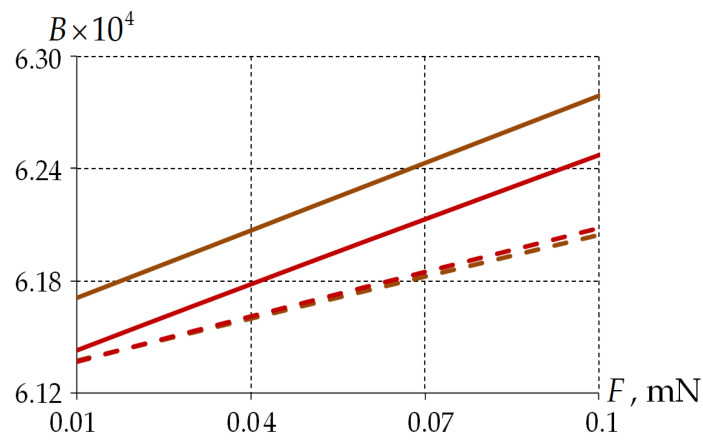
The mode birefringence’s dependence on the load: the dotted line is two-layer PC; the solid line is single-layer PC; brown line is an elastic polymer; and red line is a viscoelastic polymer.

Figure 13 shows the effect of load level on mode birefringence.

The dependence of mode birefringence on load is close to linear. The influence of polymer material behavior patterns on the optical characteristics is insignificant (less than 1%). For monolayer PCs, the birefringence under a viscoelastic polymer is less by 0.5% than under elastic PC behavior.

In the elastic glass description, the birefringence is lower by about 25% than in the viscoelastic model.

### 3.4. Analysis of the Effect of the Thickness of the Single-Layer Protective Coating on the Behavior of the Panda-Type Optical Fiber

The next step was to evaluate the effect of the thickness of the single-layer PC on the behavior of the fiber–half-space system when a constant force of 0.05 mN is applied. The coating thickness was varied from 10 to 50 µm. Similar to Section 3.2, results are shown for the viscoelastic behavior of glass under the elastic and viscoelastic behaviors of polymers.

**Figure 14:**
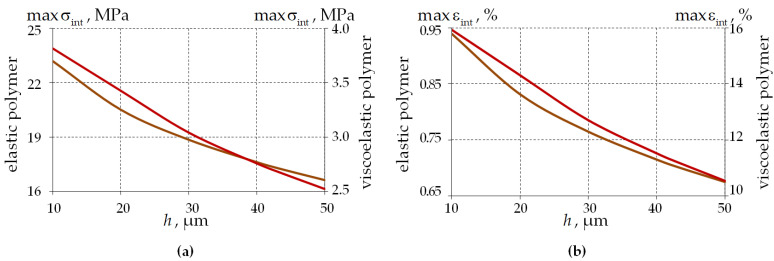
Dependence of the stress–strain state parameters in the PC2 material’s volume on the single-layer PC thickness: (**a**) the stress intensity; (**b**) the strain intensity; brown line is an elastic polymer; and red line is a viscoelastic polymer.

Dependencies of the stress–strain state parameters in the volume of the polymer coating on its thickness were obtained (Figure 14).

The level of maximum stress and strain intensity decreases with increasing coating thickness from 10 to 50 μm by 28.8 and 33.8% for elastic and viscoelastic behaviors of PCs, respectively. A nonlinear dependence of the maximum stress and strain intensity level on the thickness of the single-layer PC is observed. $\max\sigma_{\mathrm{int}}$ is on average 84.1% lower for the viscoelastic behavior of PCs. $\max\varepsilon_{\mathrm{int}}$ is on average 16.4 times higher for the viscoelastic behavior of PCs.

Similar to the previous results, the difference in the stress–strain state parameters for the elastic behavior of the glasses does not exceed 1–2%.

**Figure 15:**
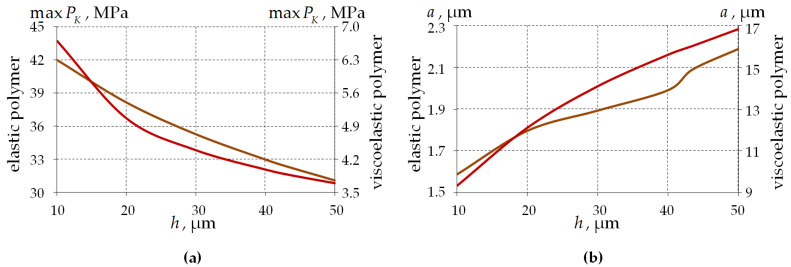
Contact parameters’ dependence on the single-layer PC thickness: (**a**) contact pressure; (**b**) the radius of the contact area; brown line is an elastic polymer; and red line is a viscoelastic polymer.

Figure 15 shows the dependence of the fiber–half-space contact area parameters on the thickness of single-layer PCs.

Similar to the stress–strain parameters, the contact pressure decreases with increasing coating thickness by 26% and 45% for elastic and viscoelastic PC behavior, respectively. The contact pressure values are on average 87.05% lower for the viscoelastic PC behavior.

The radius of the contact area increases with increased coating thickness by 37.7% and 80.1%, respectively, for elastic and viscoelastic PC behavior. The dependence of a on the layer thickness is more uniform for the viscoelastic behavior of the polymer. The value of the parameter is larger by a factor of 7.2 when comparing the viscoelastic and elastic behavior of PCs.

The glass behavior model has no influence on the parameters of the fiber and half-space contact region.

**Figure 16:**
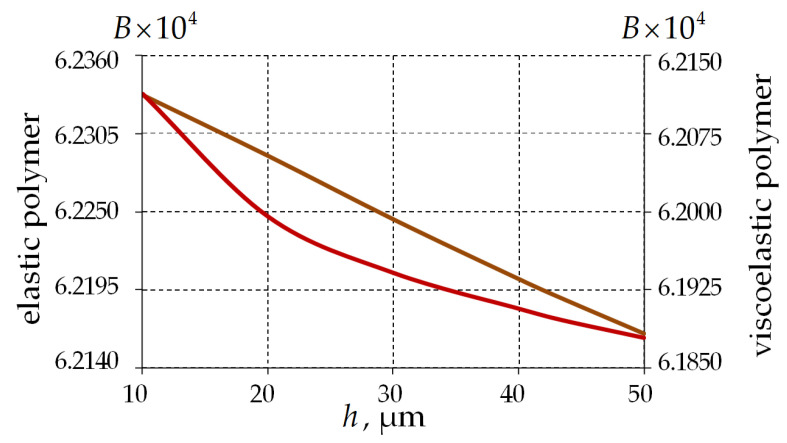
The mode birefringence’s dependence on the single-layer PC thickness: brown line is an elastic polymer; and red line is a viscoelastic polymer.

Figure 16 shows the dependence of the mode birefringence on the layer thickness.

The mode birefringence for the viscoelastic polymer is 0.5% lower than for the elastic behavior. The character of B variation is nonlinear when the viscosity of the polymer coating material is taken into account. As the thickness of the interlayer increases, B changes insignificantly (less than 0.4%), and the value of the parameter decreases.

In general, the stress–strain state of an optical fiber in a monolayer coating has insignificant design differences from a multilayer PC. Further research should consider the influence of the contact character at the interface of polymer coatings with glass and metal surfaces, and also analyze the effect of friction on system performance for different coating geometries. It is worth analyzing the effect of materials on the deformation behavior of the assembly and optics.

## 4. Discussion

### 4.1. Limitation Statement

This paper presents the results of the numerical simulation of Panda optical fiber behavior using different material behavior models for the problems of predicting residual stresses after drawing when cooling from 2000 °C to room temperature (23 °C) and indenting the fiber into an aluminum half-space at different parameters. The work has several limitations:

1. The joint deformation of the surfaces of protective coatings and quartz fiber is considered.
2. The constant coefficient of thermal expansion (CTE) of materials is used in the model. In [42], the temperature dependence of the CTE of protective coatings materials was established.
3. Models for describing the viscoelastic behavior of materials are considered: glasses are the Anand model; PC polymers are the Prony series. The dependence of material constants on temperature was not taken into account in the first approximation of the simulation.
4. The fiber has an ideal cross-sectional geometry.

Further directions for the development of the work:

1. Experimental studies of frictional properties of polymer coating materials will be conducted. Friction parameters will be refined. Experimentally determined values and dependencies of the friction coefficient at different operating modes should be introduced into the model.
2. The PC materials’ behavior during its curing with UV emitting on the technological stress fields of Panda optical fiber should be taken into account.
3. Contact interaction at the boundaries of the fiber–coating interface and between PC layers (for multilayer PC) should be considered. The influence of the interface character (ideal contact, frictional contact, full adhesion) on the behavior of the numerical fiber model should be analyzed. Influence analysis of the effect of frictional properties of PC materials for quartz–polymer, polymer–polymer and polymer–aluminum material pairs on the behavior of the fiber–PC system should be conducted.
4. Temperature variation should be accounted for in the model. The description of thermocycles should take into account real working temperatures of the study object. The influence investigation of thermocycle parameters on fiber performance at the constant coefficient of thermal expansion of protective coating polymers should be conducted.
5. The behavior model of protective coating materials should be refined by taking into account the dependence of the CTE of PC materials on temperature. Influence analysis of the effect of the refined model of material behavior on the optical and deformation characteristics of Panda optical fiber should be conducted.

### 4.2. Main Results

Cui et al. [58] note the importance of considering the viscoelasticity of silica-based optical fibers to improve and optimize their performance characteristics. The aim of their study was to evaluate the influence of fiber size (diameter) on the selection of fictitious temperatures. According to [59], fictitious temperatures characterize the transition from the liquid phase to the glassy phase. At this temperature and below, glass can be considered as an elastic body. Glass transition temperatures should be considered as a transformation range [52,60]. In [58], a formula is given to calculate the notional temperature, according to which the fictional temperature is approximately 1630 °C for a 80 µm diameter fiber. However, the formula does not take into account the chemical composition of the glass and the surface properties. In this work, it is found that at the fictitious temperature of 1600 °C the analytically determined deviation of the birefringence does not exceed 1.5%. Thus, the model of viscoelastic behavior of glass described by Smetannikov and Trufanov allows one to describe more accurately the process of cooling the fiber from the actual temperatures of the technological process by taking into account the viscosity of the material. This has been confirmed by these studies. The birefringence value of Panda fiber varies within the range of $\left[2÷5\right]\cdot 10^{-4}$ in the available literature [44,61,62,63]. The values obtained in the described multivariate study are consistent with the results of other authors. The model will allow us to increase the accuracy of calculations and proceed to modeling various optical fiber constructions [64,65,66,67]. At the same time, the results of the numerical simulation of optical fibers in the elastic formulation can be approximated to the viscosity model by means of the correction factor. It was found that, in general, the components of the stress tensor in the elastic formulation differ by 25% from those in the viscoelastic formulation. The modern capabilities of computer hardware and software make it possible to create complex mathematical models for a more accurate description of material behavior, taking into account the temperature dependences of mechanical and frictional properties.

During the drawing process, a protective coating is applied to the fiber to protect it from external influences, aggressive media, moisture and particles [68,69]. Empirical studies on the effect of the coating on the fiber’s behavior are relevant to rationalize its performance and strength [70,71]. The coating material determines the practical applications of the fiber. Single- and multilayer polymer coatings are widely used [44,72]. However, many studies do not consider polymer coatings and their effect on fiber performance [55,62,73] or consider coatings without considering the viscoelastic nature of the materials [74,75]. The results obtained in this work show that polymer coatings do have an effect on the deformation and optical performance of fibers. Describing the polymer behavior of protective coatings in an elastic setting gives highly exaggerated results for strain and contact parameters. The geometry of the coating also has an effect on the performance of the structure. It can be concluded that protective polymer coatings need to be considered when modeling fiber performance under different conditions, taking into account the viscosity of the materials. It is also necessary to complicate the polymer behavior models by taking into account CTE and frictional properties.

Taking into account the number of temperature dependences of the material parameters of fiber is one of the further research directions to obtain a more accurate description of the structure operation.

## 5. Conclusions

This model is based on the Anand model and allows the process to be modeled at temperatures above the glass transition temperature. The elastic model of glass behavior gives adequate results in the range of transformation temperatures (fictitious temperatures).

Taking into account the viscoelastic model of polymer coating behavior allows us to obtain new data on the change in deformation and optical properties under the action of external influences on the fiber. The elastic model of the material behavior of polymer coatings gives greatly overestimated results. The geometry of the protective coatings has a significant influence on the system performance. Comparative analysis of fiber operation in single-layer and two-layer PCs has shown that a single-layer protective coating is also effective and allows the contact character to be reduced to the canonical form. More detailed analysis of the influence of both the coating thickness and the materials that can be used as a protective coating is required.

A more detailed analysis of the influence of both the geometric parameters of the coating and the materials that can be used as a protective coating is required for a better description of the design performance. Further research will focus on refining material behavior models and collecting data on the influence of system parameters and materials on fiber behavior under different influences. The data are needed to rationalize and miniaturize the design without losing functionality.

## Figures and Tables

**Table 1:** Geometric parameters.

Parameter	Value	Parameter	Value
$r_{f}$	40 μm	$r_{p}$	7.5 μm
$r_{PC1}$	65 μm	$r_{b}$	3.5 μm
$r_{PC2}$	83.5 μm	$h_{bf}=r_{p}-r_{b}$	4 μm
$r_{c}$	3 μm	l	167 μm $\left(2r_{PC2}\right)$
$l_{c}$	15 μm	h	83.5 μm $\left(r_{PC2}\right)$

**Table 2:** Thermomechanical elastic properties of materials: quartz base (mat. 1 is SiO_2_); light conductor (mat. 2 is SiO_2_-doped GeO_2_); power rod internal layer (mat. 3 is SiO_2_-doped B_2_O_3_); power rod external layer (mat. 4 is SiO_2_-doped B_2_O_3_ and P_2_O_5_); material PC1 (mat. 5); material PC2 (mat. 6); aluminum (mat. 7).

Parameter	Mat. 1	Mat. 2	Mat. 3	Mat. 4	Mat. 5	Mat. 6	Mat. 7
E, MPa	72,000	67,939	49,107	65,370	2.26	2467	68,600
v	0.170	0.168	0.203	0.181	0.498	0.350	0.340
$\alpha \cdot 10^{-6}$, K^−1^	0.500	1.055	2.675	2.886	200	50	23

**Table 3:** Intensities stress in volumes of materials PC under different material behavior models.

No. Design Case	$\max\sigma_{\mathrm{int}}$, MPa		$\max\varepsilon_{\mathrm{int}}$, %	
	Material PC1	Material PC2	Material PC1	Material PC2
dc. 1	0.378	26.731	16.727	1.084
dc. 2	0.379	26.615	16.750	1.079
dc. 3	0.650	4.223	35.961	17.657
dc. 4	0.654	4.242	36.189	17.736

**Table 4:** Maximum level of stress tensor components in glass elements of optical fiber under different material behavior models with single- and two-layer PCs.

No. Design Case	Two-Layer PC			Single-Layer PC		
	$\max\sigma_{x}$ **, MPa**	$\max\sigma_{y}$ **, MPa**	$\max\sigma_{z}$, MPa	$\max\sigma_{x}$, MPa	$\max\sigma_{y}$, MPa	$\max\sigma_{z}$, MPa
dc. 1	179.253	161.739	351.728	181.419	162.548	352.248
dc. 2	224.681	203.044	440.976	227.356	204.175	441.639
dc. 3	179.315	160.151	351.547	179.340	161.057	351.662
dc. 4	224.708	201.437	440.786	224.734	202.295	440.899

**Table 5:** Comparison of stress and strain intensities in the external polymer PC and mode birefringence for single- and two-layer PCs under different behavior materials models.

No. Design Case	Two-Layer PC			Single-Layer PC		
	Material PC2		$B\times 10^{4}$	Material PC2		$B\times 10^{4}$
	$\max\sigma_{\mathrm{int}}$ **, MPa**	$\max\varepsilon_{\mathrm{int}}$ **, %**		$\max\sigma_{\mathrm{int}}$, MPa	$\max\varepsilon_{\mathrm{int}}$, %	
dc. 1	26.731	1.084	4.929	17.434	0.707	4.976
dc. 2	26.615	1.079	6.167	17.252	0.699	6.219
dc. 3	4.223	17.657	4.932	2.678	11.188	4.952
dc. 4	4.242	17.736	6.169	2.673	11.166	6.189

## Data Availability

Data are contained within the article.
